# Endocrine Therapy of Estrogen Receptor-Positive Breast Cancer Cells: Early Differential Effects on Stem Cell Markers

**DOI:** 10.3389/fonc.2017.00184

**Published:** 2017-09-04

**Authors:** Euphemia Y. Leung, Marjan E. Askarian-Amiri, Debina Sarkar, Carole Ferraro-Peyret, Wayne R. Joseph, Graeme J. Finlay, Bruce C. Baguley

**Affiliations:** ^1^Auckland Cancer Society Research Centre, University of Auckland, Auckland, New Zealand; ^2^Molecular Medicine and Pathology Department, University of Auckland, Auckland, New Zealand; ^3^Cancer Research Center of Lyon, INSERM 1052, CNRS5286, Lyon, France; ^4^Faculty of Pharmacy, University of Lyon, Claude Bernard Lyon 1 University, Lyon, France; ^5^Molecular Biology of Tumors, Hôpital Edouard Herriot, Hospices Civils de Lyon, Lyon, France

**Keywords:** breast cancer, endocrine resistance, SOX2, Wnt signaling pathway, stem cell CD24 CD44

## Abstract

**Introduction:**

Endocrine therapy of breast cancer, which either deprives cancer tissue of estrogen or prevents estrogen pathway signaling, is the most common treatment after surgery and radiotherapy. We have previously shown for the estrogen-responsive MCF-7 cell line that exposure to tamoxifen, or deprivation of estrogen, leads initially to inhibition of cell proliferation, followed after several months by the emergence of resistant sub-lines that are phenotypically different from the parental line. We examined the early responses of MCF-7 cells following either exposure to 4-hydroxytamoxifen or deprivation of estrogen for periods of 2 days–4 weeks.

**Methods:**

Endocrine-sensitive or -resistant breast cancer cell lines were used to examine the expression of the stem cell gene *SOX2*, and the Wnt effector genes *AXIN2* and *DKK1* using quantitative PCR analysis. Breast cancer cell lines were used to assess the anti-proliferative effects (as determined by IC_50_ values) of Wnt pathway inhibitors LGK974 and IWP-2.

**Results:**

Hormone therapy led to time-dependent increases of up to 10-fold in *SOX2* expression, up to threefold in expression of the Wnt target genes *AXIN2* and *DKK1*, and variable changes in *NANOG* and *OCT4* expression. The cells also showed increased mammosphere formation and increased CD24 surface protein expression. Some but not all hormone-resistant MCF-7 sub-lines, emerging after long-term hormonal stress, showed up to 50-fold increases in *SOX2* expression and smaller increases in *AXIN2* and *DKK1* expression. However, the increase in Wnt target gene expression was not accompanied by an increase in sensitivity to Wnt pathway inhibitors LGK974 and IWP-2. A general trend of lower IC_50_ values was observed in 3-dimensional spheroid culture conditions (which allowed enrichment of cells with cancer stem cell phenotype) relative to monolayer cultures. The endocrine-resistant cell lines showed no significant increase in sensitivity to Wnt inhibitors.

**Conclusion:**

Hormone treatment of cultured MCF-7 cells leads within 2 days to increased expression of components of the *SOX2* and Wnt pathways and to increased potential for mammosphere formation. We suggest that these responses are indicative of early adaptation to endocrine stress with features of stem cell character and that this facilitates the survival of emerging hormone-resistant cell populations.

## Introduction

Endocrine therapy of breast cancer, involving either deprivation of estrogen or the administration of drugs such as tamoxifen to prevent pathway signaling, is the most common treatment of breast cancer after surgery and radiotherapy. Treatment is thought to induce progressive death of cells, commencing after 3 days, in cells expressing estrogen receptor (ER) (1), followed by the gradual emergence, over several months, of hormone-resistant tumor cell populations (2–4). These populations are associated *in vivo* with both disease relapse and increased metastasis (5–7). Two changes in cell populations might be expected following therapy: an initial adaptive response of the original population to pathway inhibition, and the emergence of drug-resistant populations with altered properties. We (3, 4, 8–11) and others (12, 13) have characterized a number of endocrine therapy-resistant populations of the MCF-7 human breast cancer cell line, but these emerge after several months of exposure to hormone therapy. Here, we have sought to investigate adaptive responses that occur within the first few weeks of exposure to hormone therapy, in order to gain insights into the mechanisms of the adaptive response, and their relationship to stable, long-term resistant phenotypes.

The proliferation of breast cancer is thought to be driven by stem cell populations (14). Stem cell character is often associated with increased expression of genes, such as *SOX2, OCT4*, and *NANOG*, which also have roles in embryonic development (15). It is also associated with increased activity of the Wnt signaling pathway, which in turn is associated with the epithelial to mesenchymal transition (16). Wnt signaling involves a complex network of effectors but it can be assessed by measuring expression of the Wnt target genes *DKK1* (*Dickkopf-1*) and *AXIN2* (17). Here, we have used the MCF-7 cell line model to investigate whether cells undergo early (adaptive) changes in expression of *SOX2, DKK1*, and *AXIN2* when they have been either treated with 4-hydroxytamoxifen or deprived of estrogen. We have also measured upregulation of these markers in a series of hormone-resistant MCF-7 cell sub-lines developed by long-term selection in previous studies (3, 4, 8–11, 18). We recently showed that *SOX2* was expressed at higher levels in estrogen receptor-positive (ER+) breast tumor tissue samples from The Cancer Genome Atlas (TCGA) data set and also in tamoxifen-resistant MCF-7 breast cancer sub-lines (19).

Early changes of stem cell markers in response to therapy may provide a basis for therapy involving inhibition of the corresponding signaling pathways. Suitable inhibitors to test this hypothesis are still under development but we have carried out preliminary studies on two candidate inhibitors. The Porcupine inhibitors IWP-2 (20) and LGK974 (21) block Wnt secretion and reduce *DKK1* and *AXIN2* expression (20, 21). LGK974 is currently under clinical investigation for antitumor (including anti-breast cancer) efficacy (Trial NCT01351103). We have investigated, first, whether these drugs selectively inhibit the proliferation of hormone-resistant MCF-7 sub-lines and, second, whether the drug sensitivity correlates with the expression of the Wnt target genes *DKK1* and *AXIN2*.

## Materials and Methods

### Cell Lines and Culture Conditions

The MCF-7 human breast cancer line (22) was chosen because it is ER-positive and has been studied extensively by others as well as by us. The MCF-7 (parental) line was purchased from the ATCC, grown in alpha-MEM containing 5% fetal calf serum (FCS), penicillin/streptomycin (100 U/ml and 100 µg/ml), and insulin/transferrin/selenium supplement (Roche). We have generated tamoxifen-resistant (sub-lines TamR3, TamR6) and fulvestrant-resistant (FulvR1a, FulvR1c, FulvR2a, and FulvR3a) MCF-7 sub-lines by growing the parental cells in 1,000 nM tamoxifen or 100 nM fulvestrant in estrogen-deprived medium (EDM) (phenol red-free RPMI 1640 with 5% charcoal-stripped fetal bovine serum) and penicillin/streptomycin (100 U/ml and 100 µg/ml) for over 12 months. The sub-line TamR7 was grown in tamoxifen-supplemented complete medium with normal serum. Long-term estrogen-deprived MCF-7 sub-lines (TamC3, TamC6, FulvC1a, FulvC2, FulvC3, and FulvC4) were generated as vehicle controls by exposure to 0.1% ethanol in the same growth medium in the absence of fulvestrant or tamoxifen for the same duration. MCF-7 tamoxifen-resistant sub-lines were generated as previously described (3).

These MCF-7 sub-lines have been derived by selective culture for a prolonged period of time (over 12 months) in the presence (three sub-lines FulvR1c, FulvR2a, and FulvR3a) of fulvestrant, a “pure” anti-estrogen (4). The other five endocrine-resistant MCF-7-derived cell lines (TamR7, TamC3, TamR3, TamC6, and TamR6) have been extensively characterized (3, 4, 8–11, 18, 19, 23). Surprisingly, all of the emerging fulvestrant-treated sub-lines showed the triple negative (ER− progesterone receptor− HER2−) phenotype (4) (Figure S1 in Supplementary Material). Breast cancer cell lines (T47D, SKBR3, HCC70, MDA-MB-231, MDA-MB-468, BCC1143, and BT20) were cultured according to ATCC recommendations or as described previously (24).

For short-term tamoxifen treatment, cells were grown in alpha-MEM containing 5% FCS, penicillin/streptomycin (100 U/ml and 100 µg/ml), and insulin/transferrin/selenium supplement (Roche), and incubated with 4-hydroxytamoxifen (100 nM) for 2 days–1 month.

### Short Tandem Repeat Profiling

The relationship between the MCF-7-derived cell lines and the parental line was established using the PCR Amplification kit that amplifies 15 tetranucleotide repeat loci plus the amelogenin gender-determining marker, performed by DNA Diagnostics laboratory (Auckland, New Zealand). The combination of markers selected was consistent with the National Institute of Standards and Technology database recommendations for identity testing.

### Reverse Transcription, cDNA Synthesis, and Quantitative PCR and PCR

As described in detail previously (19), oligo-dT and random primers were used to reverse transcribe RNA with qScript Flex cDNA kit (Quantabio) according to the manufacturer’s instructions. To investigate whether increased Wnt pathway activation led to increased sensitivity to drugs that affected the Wnt pathway, quantitative RT-PCR (qRT-PCR) was performed using gene-specific primers (Table S1 in Supplementary Material) and Sybr Green MasterMix (Life Technologies), and expression values were normalized relative to *GAPDH* and *HPRT* RNA expression.

### Cell Proliferation Assay

As described in detail previously (10), cell proliferation was measured by the degree of incorporation of ^3^H-thymidine into DNA of S-phase cells. Briefly, 3,000 cells per well were seeded in 96-well plates that were tissue culture-treated for monolayer culture and incubated for 3 days. Alternatively, 6,000 cells per well were seeded in 96-well plates (Corning Costar Ultra-Low attachment) for 3 days spheroid culture. ^3^H-thymidine (0.04 μCi per well for monolayer culture or 0.08 μCi per well for spheroid culture) was added (5 h for monolayer culture or 7 h for suspension culture) prior to harvest.

### Mammosphere Formation

For mammosphere formation efficiency, MCF-7 cells in monolayer culture were exposed to 4-hydroxytamoxifen (100 nM) or solvent for 2 days, trypsinized and seeded as cell suspensions in 96-well plates coated with poly(2-hydroxyethyl methacrylate) (polyHEMA; to prevent cell attachment) (25), with 1,000 cells per well in six replicates per experiment. Mammospheres were counted after 6 days.

For mammosphere morphology and size, MCF-7 control or 4-hydroxytamoxifen-incubated cells (2 days) were trypsinized from the monolayer culture, and cell suspensions were seeded in 96-well plates (Corning Costar Ultra-Low attachment) with 2,000, 1,000 or 500 cells per well. After 4-day incubation, images were captured using FLoid Cell Imaging Station (ThermoFisher Scientific) (460× magnification). Representative images are shown. All experiments were performed at least two times.

### Flow Cytometric Analysis

Cells were grown in 25 cm^2^ culture flasks and incubated with 100 nM 4-hydroxytamoxifen for 2 days. Cells (10^6^ cells) were harvested, washed with blocking buffer [1% FCS/phosphate-buffered saline (PBS)], and incubated with 0.4 ml of antibody to CD24 (APC-H7 CD24, BD Pharmingen) or CD44 (APC CD44, BD Pharmingen) in blocking buffer (1:400 dilution) on ice for 30 min. Cells were washed and resuspended in 0.5 ml of blocking buffer. Cells were analyzed in a BD Accuri™ Flow Cytometer and profiles were analyzed with FlowJo V10 software.

### Viability Assay

Mitochondrial activity was measured by the Alamar Blue assay (Life Technologies). Superoxide dismutase was measured using a water-soluble tetrazolium salt (WST-1) (purchased from Roche) (26). The stable tetrazolium salt WST-1 is cleaved to a soluble formazan by a complex cellular mechanism that occurs primarily at the cell surface. This bioreduction is largely dependent on the glycolytic production of NAD(P)H in viable cells. Therefore, the amount of formazan dye formed directly correlates with the number of metabolically active cells in the culture. All experiments were done in triplicate wells and performed at least two times.

### Immunoblotting

As described in detail previously (10), breast cancer cell lines were grown to log-phase, washed twice with ice-cold PBS, and lysed in sodium dodecyl sulfate (SDS) lysis buffer according to the manufacturer’s protocol (Cell Signaling Technology, Danvers, MA, USA). Protein concentration was quantified using the bicinchoninic acid reagent (Sigma). Cell lysates containing 25 µg of protein were separated by SDS-polyacrylamide gel electrophoresis, and transferred to PVDF membranes (Millipore). Membranes were immunoblotted with antibodies against SOX2 (Cell Signaling Technology) and tubulin (Sigma). Bound antibody was visualized using SuperSignal West Pico (Thermo Scientific, Waltham, MA) or ECL plus (GE Healthcare, Auckland, New Zealand) and the chemiluminescence detection system by Fujifilm Las-3000.

### Data Analysis

*T*-test or Mann–Whitney rank sum test was used for comparison between two groups. Correlation analysis was performed with Spearman’s rank order correlation coefficient (R) and statistical significance (P) using SigmaPlot (Systat Software, Inc.). Values of *p* < 0.05 were considered to be statistically significant.

## Results

### Expression of *SOX2, AXIN2*, and *DKK1*: An Early Response to Endocrine Therapy in the MCF-7 Breast Cancer Cell Line

**Figure 1:**
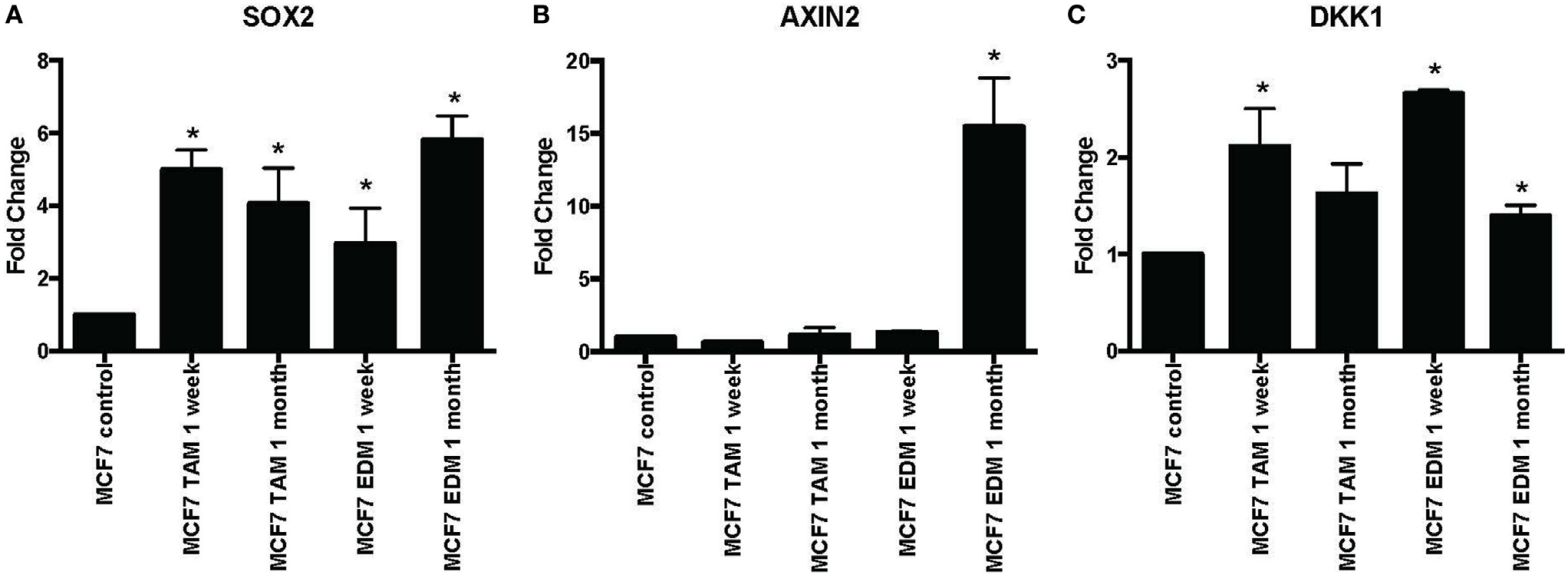
Increased expression of *SOX2, AXIN2*, and *DKK1* in MCF-7 breast cancer cells in 4-hydroxytamoxifen-supplemented or estrogen-deprived medium (EDM). The expression of **(A)** *SOX2*, **(B)** *AXIN2*, and **(C)** *DKK1* mRNA relative to that of MCF-7 control was measured by quantitative RT-PCR in MCF-7 breast cancer cells either exposed to 4-hydroxytamoxifen (TAM; 100 nM) or grown in EDM for 1 week and 1 month, relative to the MCF-7 parental line. Error bars represent the SEM of three biological replicates, *p < 0.05.

*SOX2* expression was found to be significantly increased when MCF-7 cells were either exposed to 4-hydroxytamoxifen or grown in EDM for up to 1 month (Figure 1A). Expression of the Wnt target genes *AXIN2* and *DKK1* was also investigated, since they were reported to be significantly upregulated in stably MCF-7 tamoxifen-resistant MCF-7 cells (>4 months selection) that overexpressed *SOX2* (12). *AXIN2* was significantly increased when MCF-7 cells were grown in EDM for 1 month and *DKK1* was also significantly increased in the treatment group after 1 week (Figures 1B,C).

**Figure 2:**
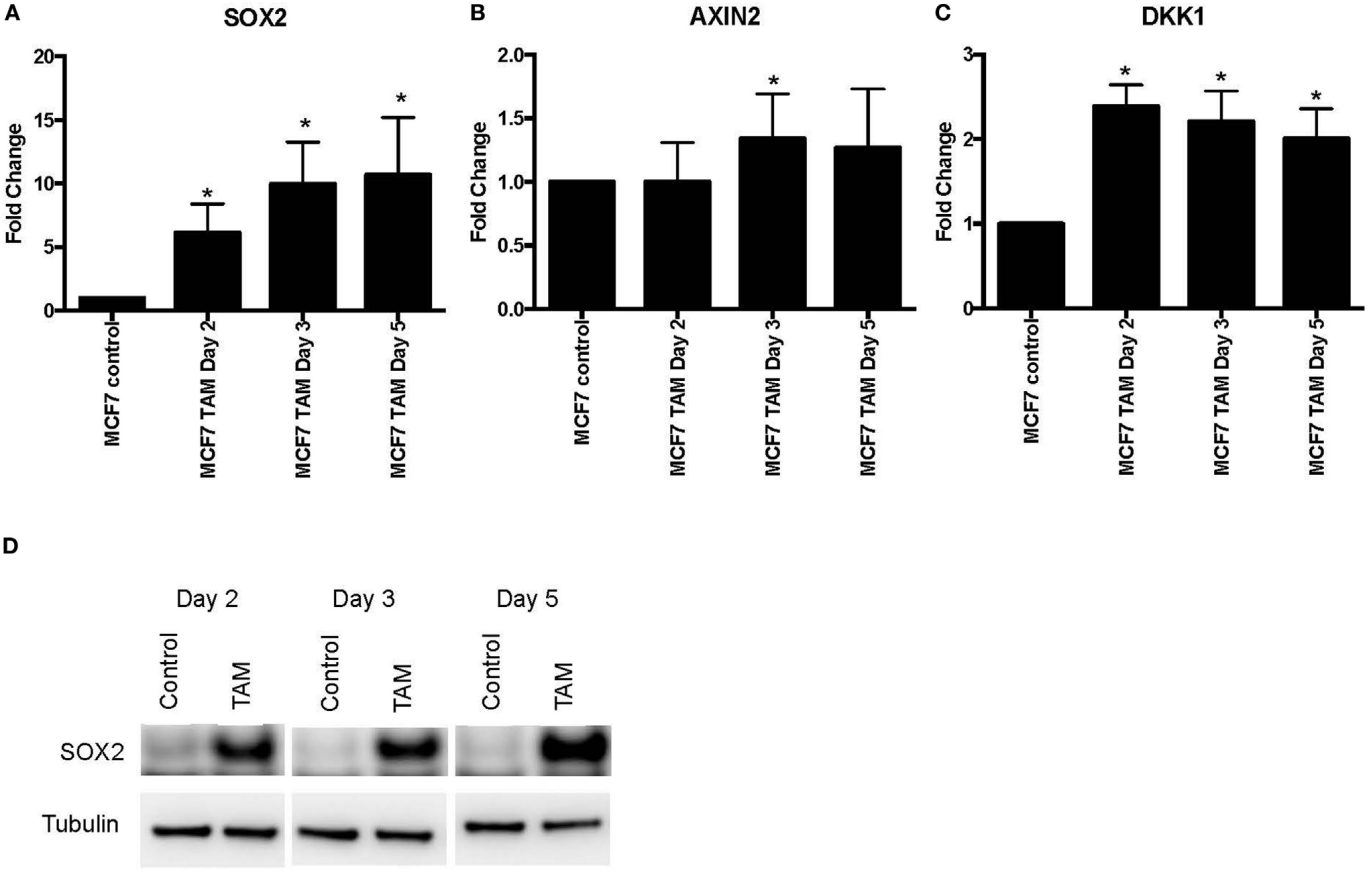
Increased expression of *SOX2, AXIN2*, and *DKK1* in MCF-7 breast cancer cells with short-term 4-hydroxytamoxifen treatment. The expression of **(A)** *SOX2*, **(B)** *AXIN2*, and **(C)** *DKK1* relative to MCF-7 control measured by quantitative RT-PCR in MCF-7 breast cancer cells exposed to 4-hydroxytamoxifen (100 nM) for 2–5 days, relative to the MCF-7 parental line. **(D)** Western blot analysis showing expression of SOX2 in 4-hydroxytamoxifen (TAM; 100 nM)-treated MCF-7 cells. Tubulin is the loading control.

We also examined earlier responses to hormone therapy of MCF-7 cells. The expression of *SOX2, AXIN2*, and *DKK1* was determined in MCF-7 cells exposed to 4-hydroxytamoxifen for 5 days or less. As expected, both *SOX2* and *DKK1* were significantly upregulated in tamoxifen-treated cells, while *AXIN2* showed a general trend toward upregulation with tamoxifen treatment (Figures 2A–C). SOX2 protein was also induced in parallel with the upregulated *SOX2* mRNA expression (Figure 2D). We also investigated whether *NANOG* and *OCT4* were differentially modulated during the development of tamoxifen resistance (Figure S2 in Supplementary Material). *OCT4* expression appeared to increase significantly at the later time (1 month) with tamoxifen exposure, while *NANOG* showed significant increase in 1 week of 4-hydroxytamoxifen exposure and also when grown in EDM for 1 month. However, expression levels of *SOX2, NANOG*, and *OCT4* were not correlated (Table S2 in Supplementary Material).

### Expression of Stem Cell Character in Response to Endocrine Therapy in the MCF-7 Breast Cancer Cell Line

**Figure 3:**
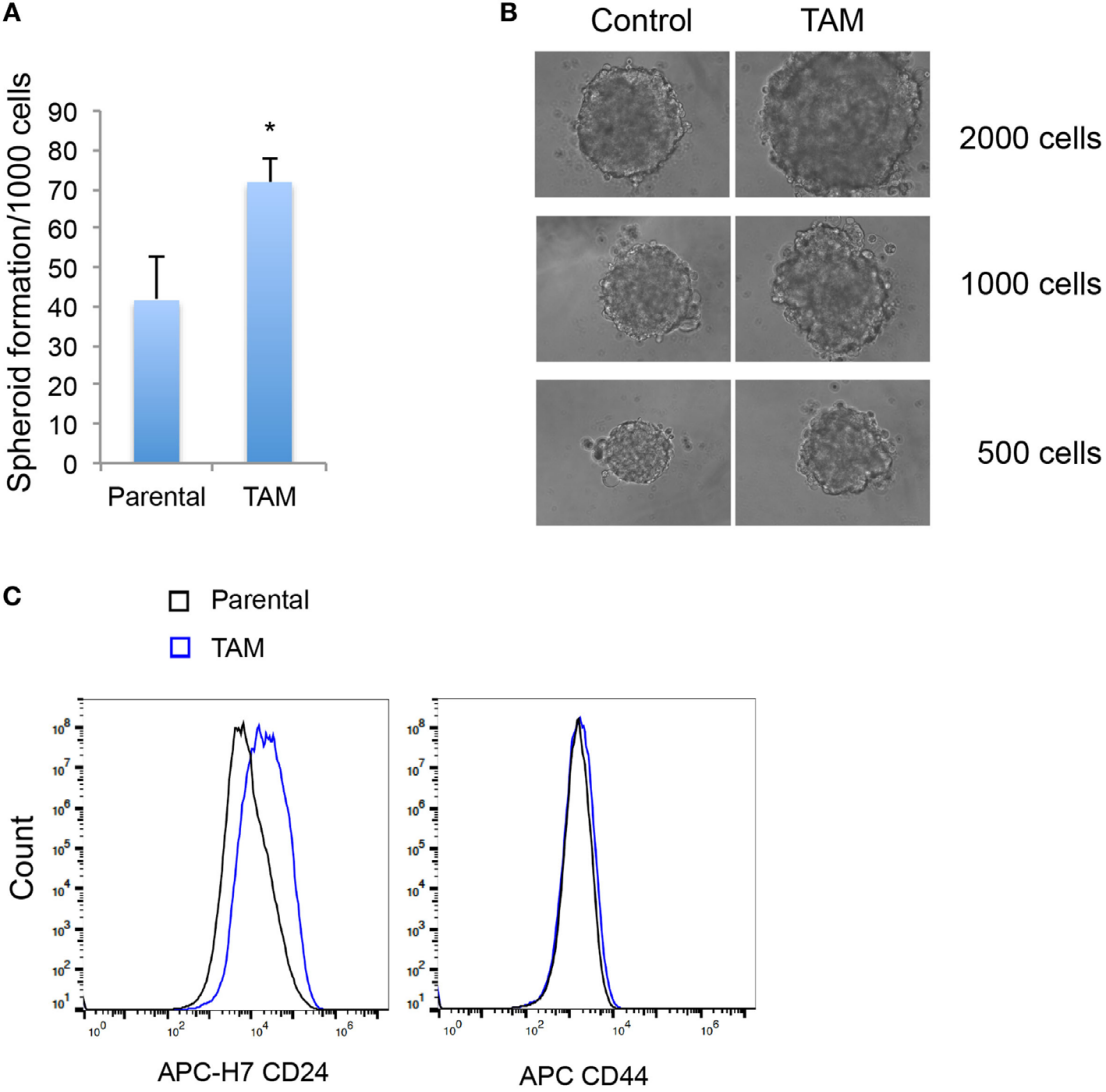
**(A)** Mammosphere formation efficiency of MCF-7 cells exposed to 4-hydroxytamoxifen relative to that of vehicle-exposed cells. **(B)** Mammosphere morphology of MCF-7 cells exposed to 4-hydroxytamoxifen (TAM; 100 nM) compared with the MCF-7 parental line. **(C)** Increased expression of CD24, but not CD44, in MCF-7 cells exposed to 4-hydroxytamoxifen (100 nM) for 2 days. *Significantly different from MCF-7 parental line (*p* < 0.05). Error bars represent the SEM of three biological replicates, *p < 0.05.

We next examined the stem-like character (mammosphere formation efficiency) as an early response to tamoxifen exposure in MCF-7 cells. As expected, the increase of *SOX2* expression was associated with an increased ability to form mammospheres (Figures 3A,B). An increase in CD24, but not in CD44, expression was also detected in MCF-7 cells exposed to 4-hydroxytamoxifen for 2 days (Figure 3C).

### Expression of *SOX2, AXIN2*, and *DKK1* in Endocrine-Resistant MCF-7 Breast Cancer Cells

**Figure 4:**
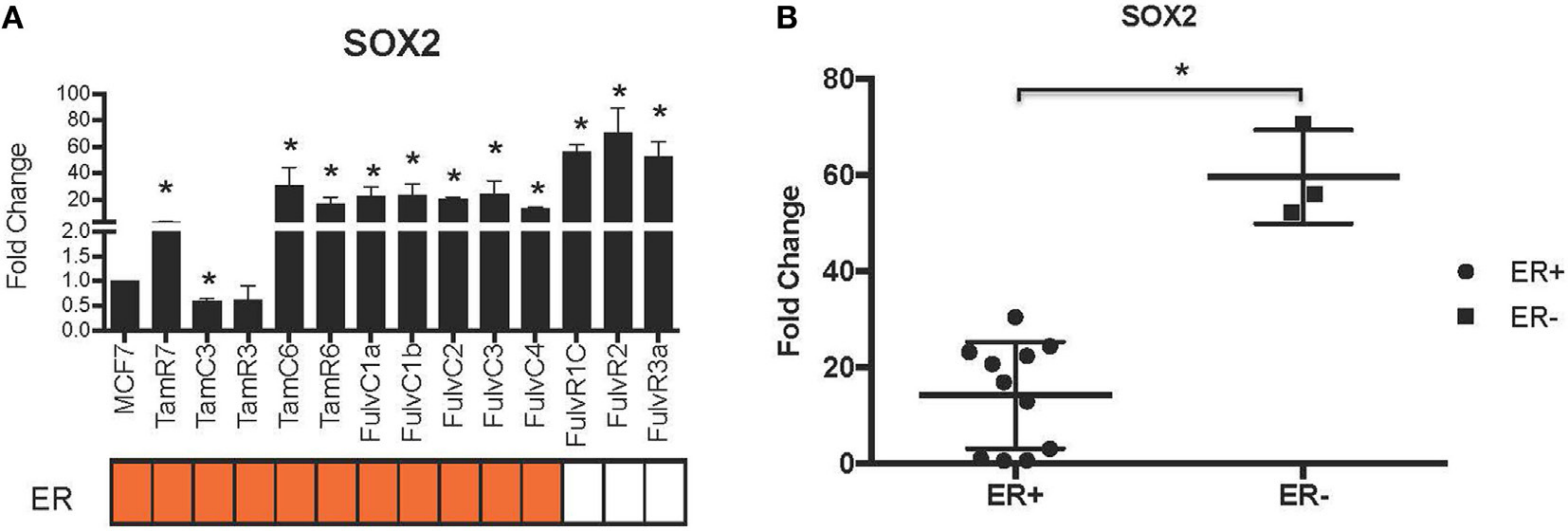
Increased expression of *SOX2* in 13 MCF-7 endocrine therapy-resistant sub-lines. **(A)** The expression of *SOX2* relative to MCF-7 control measured by quantitative RT-PCR in MCF-7 breast cancer cells with either long-term exposure to tamoxifen or fulvestrant, or growth in estrogen-deprived medium. **(B)** Box plot indicating expression of *SOX2* in estrogen receptor (ER+) and ER− MCF-7 breast cancer sub-lines. Error bars represent the SEM of three biological replicates, *p < 0.05.

**Figure 5:**
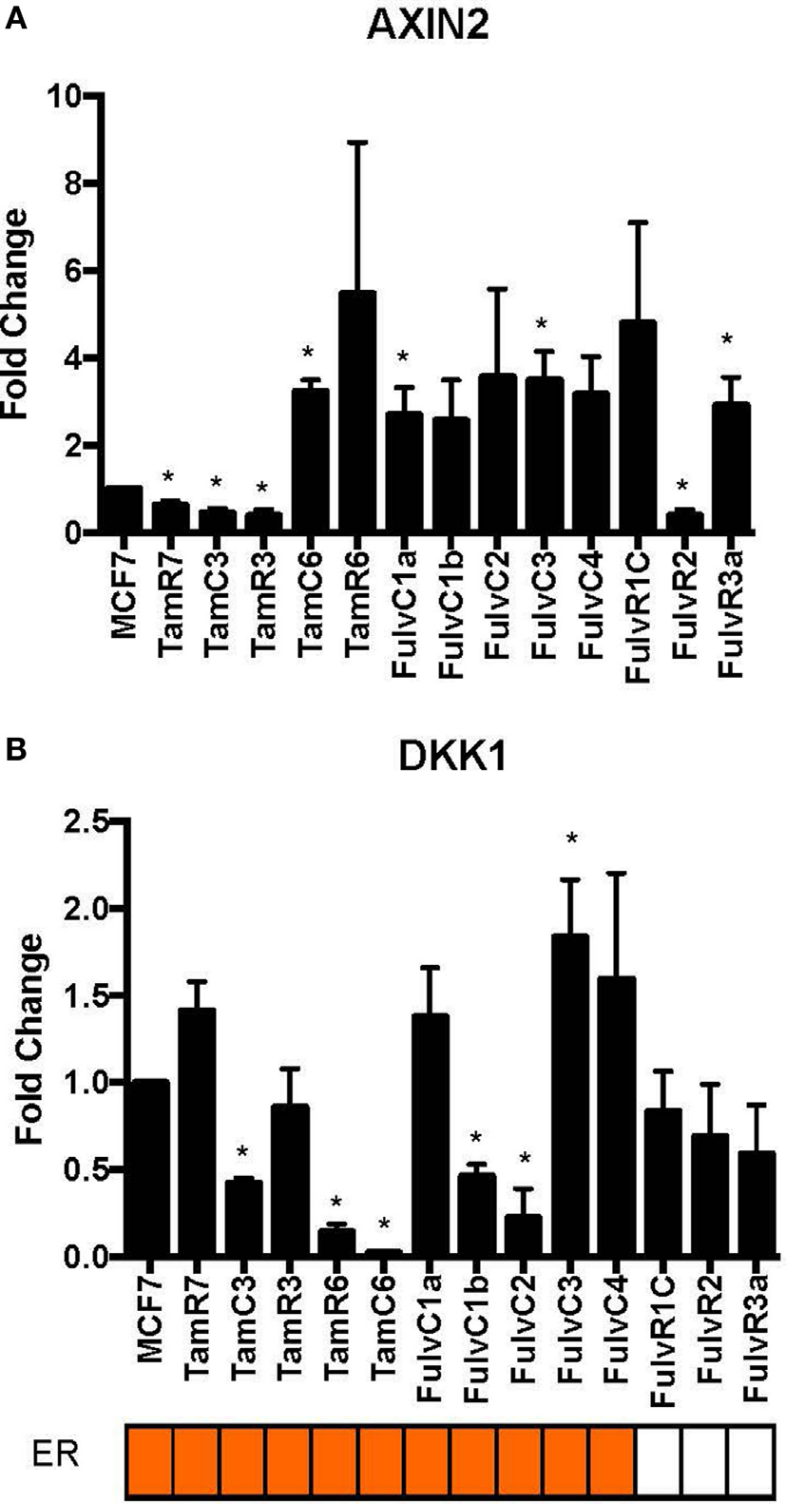
Variations in expression of *AXIN2* and *DKK1* in 13 MCF-7 endocrine therapy-resistant sub-lines. The expression of **(A)** *AXIN2* and **(B)** *DKK1* relative to MCF-7 control measured by quantitative RT-PCR in MCF-7 breast cancer cell lines with either long-term exposure to tamoxifen or fulvestrant, or growth in estrogen-deprived medium. Error bars represent the SEM of three biological replicates, *p < 0.05.

Using our panel of endocrine therapy-resistant breast cancer cell lines generated by long-term estrogen-deprived culture conditions, or anti-estrogen treatment (3, 4, 8–11, 18, 27), we examined the gene expression pattern of *SOX2*, together with the WNT effector genes *DKK1* and *AXIN2*, to assess long-term expression changes of these stem cell markers. All except two endocrine therapy-resistant breast cancer lines showed significant upregulation of *SOX2* expression (Figure 4A). The ER− endocrine-resistant breast cancer lines (generated by fulvestrant treatment) showed significant upregulation of *SOX2* expression as compared to the ER+ cell lines (Figure 4B). Four resistant sub-lines showed a significantly increased *AXIN2* expression (Figure 5A). Although there was a general trend of reduction of *DKK1* expression, only five sub-lines showed significant reduction in *DKK1* expression and one showed a significant increase in *DKK1* expression (Figure 5B). Contrary to the published data (12), we found that in the MCF-7 endocrine therapy-resistant sub-lines, the expression levels of *SOX2, DKK1*, and *AXIN2* were not correlated (Table 1). In addition to the MCF-7 cell lines, we used seven additional breast cancer cell lines to compare the expression of *SOX2* and *AXIN2* or *DKK1* but no significant correlations were found (Table S3 in Supplementary Material).

**Table 1:** Spearman’s rank order correlation of relative gene expression values for *SOX2, AXIN2*, and *DKK1* in MCF-7 parent and endocrine therapy-resistant sub-lines (*n* = 14).

	*AXIN2*	*DKK1*
*SOX2*	0.43	−0.12
*p*-Value	0.12	0.68
*AXIN2*		−0.30
*p*-Value		0.28

### Correlation of Stem Cell Genes *SOX2* and Wnt Pathway Genes *AXIN2* and *DKK1* in Breast Cancer Samples from TCGA Data Set

We examined the correlation of the expression of *SOX2* and Wnt pathway effector genes *AXIN2* and *DKK1* in the genome-wide RNA transcript profile from TCGA (breast invasive carcinoma gene expression) using the RNAseq data set (TCGA_BRCA_exp_HiSeqV2-2015-02-24) in 1,009 tumor tissue samples from breast cancer patients. A weak but significant negative correlation was observed in the expression of *AXIN2* and *DKK1* (Spearman’s rank order correlation coefficient, *r* = −0.15, *p* = 2 × 10^−6^). Expression of *SOX2* and *AXIN2* or *DKK1* was not significantly correlated (Table 2). We next stratified the dataset into the ER+ and ER− breast cancer samples, but no correlation was found between *SOX2* and *AXIN2* or *DKK1*, or between *AXIN2* and *DKK1* expression in ER+ (Table S4 in Supplementary Material) or ER− samples (Table S5 in Supplementary Material).

**Table 2:** Spearman’s rank order correlation coefficients of relative gene expression values for *SOX2, AXIN2*, and *DKK1* in breast cancer samples from The Cancer Genome Atlas (TCGA_BRCA_exp_HiSeqV2-2015-02-24) data set (*n* = 1,009), *p < 0.05.

	*AXIN2*	*DKK1*
*SOX2*	0.01	−0.027
*p*-Value	0.7	0.4
*AXIN2*		−0.15
*p*-Value		0.000002*

### Responses in a Panel of MCF-7 Breast Cancer Sub-Lines to Wnt Pathway Inhibitors LGK974 and IWP-2 under Monolayer and Spheroid Culture Conditions

**Figure 6:**
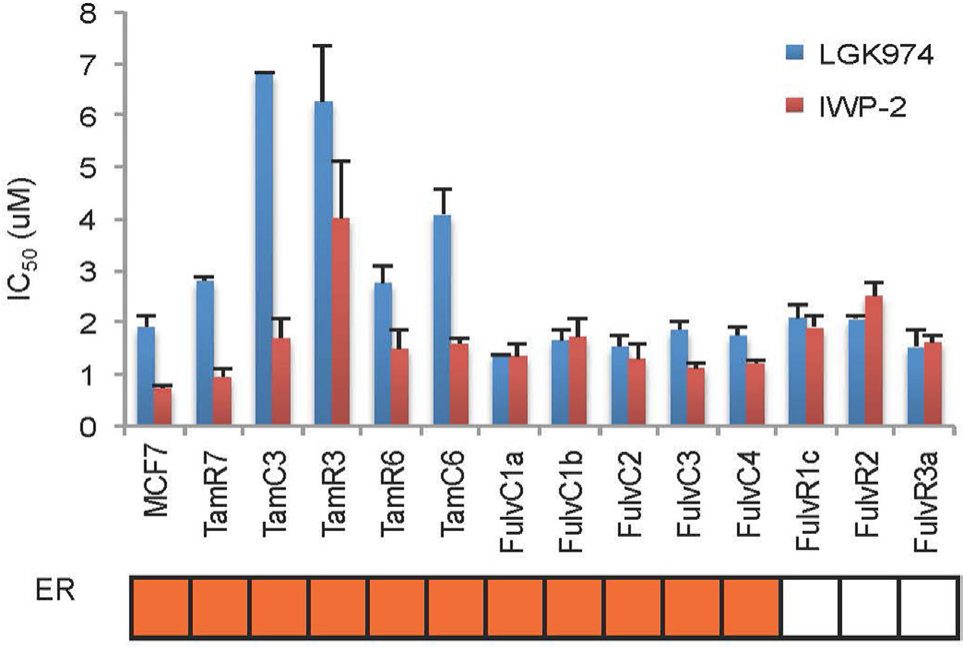
Comparison of LGK974 and IWP-2 drug sensitivity of breast cancer endocrine-resistant cell lines. IC_50_ values (50% inhibition of thymidine incorporation relative to non-drug-treated cells) for LGK974 and IWP-2 are represented on the *y*-axis and individual cell lines on the *x*-axis. Cell lines are colored orange for estrogen receptor-positive status (ER). Results are shown as the mean ± SE from triplicate experiments. IC_50_ values for LGK974 and IWP-2 were not significantly correlated (Spearman’s rank correlation coefficient, *r* = 0.28; *p* = 0.33).

An endocrine therapy-resistant MCF-7 cell line has been reported to be sensitive to Wnt inhibitors (12). The Wnt pathway inhibitors LGK974 and IWP-2 inhibit palmitoylation of Wnt ligands, thus blocking Wnt secretion (20, 21). MCF-7 and its endocrine therapy-resistant sub-lines were assayed for sensitivity to LGK974 and IWP-2 (as measured by IC_50_ values) (Figure 6). Endocrine-resistant cell lines did not show increased sensitivity to Wnt inhibitors as compared to parental cell line. In MCF-7 parental and endocrine therapy-resistant sub-lines, the IC_50_ values of the Wnt inhibitors LGK974 (range from 1.4 to 6.8 µM) and IWP-2 (0.7–4 µM) were not significantly correlated (Spearman’s rank order correlation coefficient, *r* = 0.28, *p* = 0.33). Neither LGK974 nor IWP-2 (10 µM) reached the IC_50_ with any cell line, using the WST-1 and Alamar Blue viability assays (Figure S3 in Supplementary Material).

**Figure 7:**
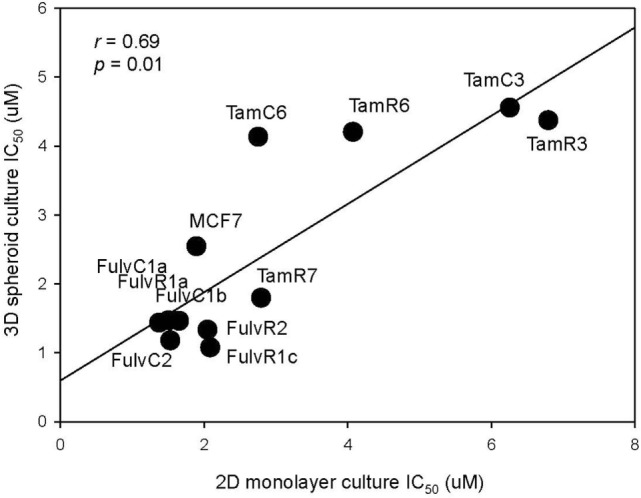
Correlation of drug sensitivity in monolayer cultures and in three dimension (3D) spheroid cultures. Plot of IC_50_ values for LGK974 in 2D monolayer culture (*x*-axis) and 3D spheroid culture (*y*-axis). Experiments were performed in triplicate wells and performed twice (Spearman’s rank correlation coefficient, *r* = 0.69, *p* = 0.01).

As three dimension (3D) spheroid culture conditions allowed enrichment of cancer cells with a cancer stem cell phenotype, we next examined the sensitivities of our MCF-7 sub-lines to the LGK974 inhibitor in 3D spheroid culture and compared them to the sensitivities of the same cells grown in adherent monolayer culture conditions. Although there was no significant difference (*T* test, *p* > 0.05) in the IC_50_ of the 2D monolayer (range from 1.5 to 6.3 µM) and 3D spheroid culture conditions (range from 1.1 to 4.4 µM), a general trend of lower IC_50_ was observed in 3D spheroid culture conditions (Figure 7). The IC_50_ values arising from the two culture conditions were positively correlated (Spearman’s rank order correlation coefficient, *r* = 0.69, *p* = 0.01).

We next examined whether the expression levels of *SOX2, AXIN2*, or *DKK1* were associated with altered drug sensitivity of the MCF-7 endocrine therapy-resistant sub-lines to LGK974. Expression of *SOX2, AXIN2*, and *DKK1* and the growth inhibitory effects (IC_50_ value) of the Wnt inhibitor LGK974 were not significantly correlated (Table 3).

**Table 3:** Spearman’s rank order correlation coefficients of relative gene expression values (fold change) for *AXIN2, DKK1*, and *SOX2* versus IC_50_ value of the WNT inhibitors LGK974 and IWP-2 in MCF-7 parental and endocrine therapy-resistant breast cancer cell lines (*n* = 14).

	*SOX2*	*AXIN2*	*DKK1*
LGK974 (*r*^2^)	−0.43	−0.26	−0.23
*p*-Value	0.12	0.36	0.42
IWP-2 (*r*^2^)	0.28	−0.25	−0.42
*p*-Value	0.31	0.37	0.13

## Discussion

The principal finding of this study is that it demonstrated the difference between early responses (Figures 1–3) and late stable phenotypes (Figures 4–7) following endocrine treatment of MCF-7 cells. Either exposure of cultured MCF-7 cells to 4-hydroxytamoxifen or depletion of estrogen from the growth medium leads after 7 days to threefold to sixfold increases in *SOX2* expression. Increases are highly reproducible, evident within 2 days and persist for at least 1 month (Figures 1 and 2). These changes are accompanied by a decrease in proliferation rate, as shown by others (28). Longer term exposure to tamoxifen, or to estrogen deprivation, leads after several months to the emergence of discrete hormone-resistant subpopulations, distinguishable from the hormone-sensitive line by cellular DNA content and other properties (3, 4). Most but not all of these emerging populations continue to over-express *SOX2* (Figure 4) and it was notable that the two lines (TamC3 and TamR3) that did not exhibit increased SOX2 expression instead showed increased PAX2 signaling (3). We hypothesize that upregulation of *SOX2* expression is part of a stress response generated by endocrine therapy.

Several observations suggest that the stress response is associated with an increase in stem cell-like properties of the treated population. SOX2 is a recognized marker of stem cell properties (29) and we have found that components of the WNT pathway, which are also associated with stem cell-like properties (30), are increased in MCF-7 cells exposed to endocrine therapy. Significant increases in the Wnt target genes *DKK1* and *AXIN2* expression followed estrogen deprivation. Expression of *DKK1* was increased transiently between 2 and 7 days (Figures 1 and 2) but not after 1 month of endocrine treatment. Some but not all sub-lines of MCF-7 emerging after endocrine therapy maintained upregulation of *AXIN2* and *DKK1* (Figure 5). *SOX2* expression has previously been reported to be associated with increased expression of the Wnt effector genes *AXIN2* and *DKK1* (12), although in our analysis of TCGA tumor tissue samples, the expression levels of *AXIN2* or *DKK1* were not significantly correlated with those of *SOX2*. This lack of correlation suggests that the *SOX2* and Wnt signaling pathways may be partially independent of each other; Wnt induction of *AXIN2* and *DKK1* may form negative feedback loops that dampen Wnt signaling, reducing the functional linkage to Wnt pathway inhibitor sensitivity (17, 31). The upregulation of *AXIN2* expression in our endocrine therapy-resistant cell lines is in agreement with the findings of others (32). Expression of stem cell genes *OCT4* and *NANOG* (transcription factors essential for self-renewal and pluripotency maintenance of stem cells) (33) was also increased in MCF-7 cells exposed to endocrine therapy as an early response. Increased efficiency of mammosphere formation and increased expression of the surface protein CD24 add further evidence in favor of the hypothesis that the MCF-7 breast cancer cells acquire stem cell-like properties as an early response to endocrine therapy. The increase in CD24 was consistent with the observation that estrogen mediated downregulation of CD24 in breast cancer cells (34). Knockdown of CD24 by siRNA is known to inhibit cell viability and invasion, to induce apoptosis, and to increase the sensitivity of MCF-7 cells to tamoxifen (35, 36).

The results with the MCF-7 cell line may provide insights into possible *in vivo* events in breast cancer patients undergoing hormone therapy. SOX2 levels are elevated in primary tumors of patients who do not respond to endocrine therapy (12), and SOX2 has been reported to maintain stem cell subpopulations in various cancers (37). Another report concludes that estrogen reduces the proportion of breast stem cells while tamoxifen increases it (38), consistent with our data. SOX2 overexpression has been correlated with breast cancer tumor grade (39), suggesting that it contributes to tumor aggression. SOX2 has also been implicated in endocrine resistance in breast cancer (12) and in the development of chemo-resistance in gastric cancer (40), glioblastoma (41), head and neck squamous cell carcinoma (42), lung cancer (43), and prostate cancer (44). Dysregulated Wnt signaling is observed in breast cancer (45, 46) and Wnt signaling has been reported to be an early event in a breast neoplasia model (47). The Wnt effector AXIN2 is upregulated in breast carcinoma (48, 49) and is involved in the Wnt-Axin2-GSK3beta cascade to regulate the epithelial–mesenchymal transition in breast cancer cells (50). Dickkopf-1 (DKK-1) is preferentially expressed in tumors with poor prognosis, ER− breast cancer and endocrine therapy-resistant tumors (51). Overexpression of DKK1 has also been reported in many cancers, including breast cancer, prostate cancer, lung cancer, and multiple myeloma (52–54). Activation of the Wnt pathway improves survival of human breast cancer cells and rescues ER+ tumor cells from tamoxifen (55).

In fact, CD44−/CD24+ is a marker of poor prognosis in early invasive breast cancer (56). CD24 overexpression has also been related to shorter distant metastasis-free survival in breast cancer patients (57). A proposed cancer stem cell model anticipates hierarchical organization but also constant clonal evolution of tumor cells (33). It is, therefore, plausible that early adaptive responses to tamoxifen favor the generation of CD44−/CD24+ cells and that continued therapy leads to stable phenotypes of CD44+/CD24− stem cell as reported (12). Alternatively, there may be technical differences as charcoal-stripped serum was used for our selection for stable phenotypes.

The above results suggest that targeting of the SOX2 and Wnt pathways might inhibit the emergence of endocrine-resistant breast cancer cells. We did not have inhibitors of SOX2 but did carry out preliminary studies on the inhibitory effects of the Porcupine inhibitors IWP-2 and LGK974, which target the Wnt pathway. LGK974 has been reported to inhibit Wnt secretion and reduce the expression of the Wnt target gene *AXIN2* (21). The abilities of IWP-2 and LGK974 to inhibit cell proliferation were tested but the resulting IC_50_ values were not significantly correlated (Figure 6). We also measured the effect of LGK974 on MCF-7 cell lines in 3D spheroid culture, as compared to 2D monolayer culture and found significant correlation of the IC_50_ values (*r* = 0.69, *p* = 0.01), suggesting that further study of the Wnt pathway may be warranted as a therapeutic strategy. Alternatively, if there is a hierarchy of stem cells, Wnt inhibition may affect different signaling pathways at different times.

In conclusion, the large changes in expression of *SOX2* in response to *in vitro* endocrine therapy, together with smaller changes in components of the Wnt pathway, especially those that are early in the response to 4-hydroxytamoxifen, suggest that they could be involved as an intrinsic part of the transition to hormone resistance. We suggest that a new state with increased stem cell-like character is induced and promotes phenotypic diversification such that the discrete emerging sub-lines show varying degrees of ER expression, EGFR expression, and mTOR pathway utilization (3). Further research on the mechanisms underlying such changes is warranted.

## Author Contributions

EL and BB designed this study. EL, MA-A, DS, CF-P, and WJ carried out the study. EL, CF-P, and MA-A assisted with the data analysis. EL drafted the manuscript. GF and BB assisted with the manuscript preparation. All the authors read and approved the final manuscript.

## Conflict of Interest Statement

The authors declare that the research was conducted in the absence of any commercial or financial relationships that could be construed as a potential conflict of interest. The reviewer AH and handling Editor declared their shared affiliation.

## Supplementary Material

Figure S1 Relative expression of estrogen receptor (ER), progesterone receptor (PR), and HER2 in SKRB3, MCF-7 and a selection of its sub-lines. Actin is shown as loading control.

Figure S2 Increased expression of *NANOG* and *OCT4* in MCF-7 breast cancer cells in 4-hydroxytamoxifen or estrogen-deprived treatment. The expression of **(A)** *NANOG* and **(B)** *OCT4* relative to MCF-7 control measured by qRT-PCR in MCF-7 breast cancer cells either exposed to 4-hydroxytamoxifen (100 nM) or grown in estrogen-deprived medium (EDM) for 1 week and 1 month, relative to the MCF-7 parental line, **p* < 0.05.

Figure S3 Relative viability of LGK974 and IWP-2 among breast cancer endocrine-resistant cell lines. Viability was measured by **(A)** WST-1 and **(B)** Alamar blue. Results are shown as the mean ± SE from duplicate experiments.

## Abbreviations

DKK1, Dickkopf-1; AXIN2, axis inhibition protein 2; SOX2, SRY (sex determining region Y)-box 2; OCT4, octamer-binding transcription factor 4, also known as POU5F1; NANOG, NANOG homeobox; ER, estrogen receptor; PR, progesterone receptor; HER2, human epidermal growth factor receptor 2; qRT-PCR, quantitative reverse transcription polymerase chain reaction; PBS, phosphate-buffered saline; SDS, sodium dodecyl sulfate; TCGA, The Cancer Genome Atlas.
