# Supplementary material for: Endocrine Therapy of Estrogen Receptor-Positive Breast Cancer Cells: Early Differential Effects on Stem Cell Markers
Source: Front Oncol. 2017 Sep 4;7:184. doi: 10.3389/fonc.2017.00184 (PMC5591432; doi:10.3389/fonc.2017.00184)
Supplement: Supplementary file 1 [file Table_1.DOCX]

Table S1. List of primers used in these experiments.

| SOX2-F | 5’-AACCCCAGATGCACAA CTC-3’ |
| --- | --- |
| SOX2-R | 5’-GCTTAGCCTCGTCGATGAAC-3’ |
| HPRT-F | 5’-TGAGGATTTGGAAAGGGTGT-3’ |
| HPRT-R | 5’-GCACACAGAGGGCTACAATG-3’ |
| GAPDH-F | 5’-ACGGG AAGCTTGTCATCAAT-3’ |
| GAPDH-R | 5’-TGGACTCCACGACGTACTCA-3’ |
| AXIN2-F | 5’-TGATCTCGAGGCTGGAAAAG-3’ |
| AXIN2-R | 5’-CTCGGAGCCCTCTCTCTCTT-3’ |
| DKK1-F | 5’-GGGAATTACTGCAAAAATGGAA-3’ |
| DKK1-R | 5’-CCCATCCAAGGTGCTATGAT-3’ |
| NANOG-F | 5’-GTGGAGGAAGCTGACAACAA-3’ |
| NANOG-R | 5’-CTCCAGGTTGCCTCTCACTC-3’ |
| OCT4-F | 5’-CCTATGCCTGTGATTTGTGG-3’ |
| OCT4-R | 5’-CTTGACCGGGACCTTGTCTT-3’ |

Table S2. Spearman Rank Order Correlation of relative gene expression values for *NANOG*, *OCT4*, and *SOX2* in breast cancer cell lines MCF7 during the development of tamoxifen resistance.

|  |  |  |
| --- | --- | --- |
|  | ***NANOG*** | ***OCT4*** |
| SOX2 (*r*) | 0.9 | 0.9 |
| *p* value | 0.08 | 0.08 |

Table S3. Spearman Rank Order Correlation of relative gene expression values for *AXIN2*, *DKK1*, and *SOX2* in breast cancer cell lines MCF7, T47D, SKBR3, HCC70, MDA-MB-231, MDA-MB-468, HCC1143 and BT20 (n=8).

|  |  |  |
| --- | --- | --- |
|  | ***AXIN2*** | ***DKK1*** |
| SOX2 (*r*) | -0.45 | 0.52 |
| *p* value | 0.23 | 0.16 |

Table S4. Spearman Rank Order Correlation of relative gene expression values for *AXIN2*, *DKK1*, and *SOX2* in ER+ breast cancer samples from The Cancer Genome Atlas (TCGA_BRCA_exp_HiSeqV2-2015-02-24) data set (n=601).

|  | ***AXIN2*** | ***DKK1*** |
| --- | --- | --- |
| ***SOX2*** (*r*) | -0.07 | -0.009 |
| *p* value | 0.08 | 0.81 |
|  |  |  |
| ***AXIN2*** (*r*) |  | -0.03 |
| *p* value |  | 0.44 |

Table S5. Spearman Rank Order Correlation of relative gene expression values for *AXIN2*, *DKK1*, and *SOX2* in ER- breast cancer samples from The Cancer Genome Atlas (TCGA_BRCA_exp_HiSeqV2-2015-02-24) data set (n=179).

|  | ***AXIN2*** | ***DKK1*** |
| --- | --- | --- |
| ***SOX2*** (*r*) | -0.01 | 0.09 |
| *p* value | 0.88 | 0.23 |
|  |  |  |
| ***AXIN2*** (*r*) |  | -0.11 |
| *p* value |  | 0.15 |
