# Supplementary figures and images for: Endocrine Therapy of Estrogen Receptor-Positive Breast Cancer Cells: Early Differential Effects on Stem Cell Markers
Source: Front Oncol. 2017 Sep 4;7:184. doi: 10.3389/fonc.2017.00184 (PMC5591432; doi:10.3389/fonc.2017.00184)

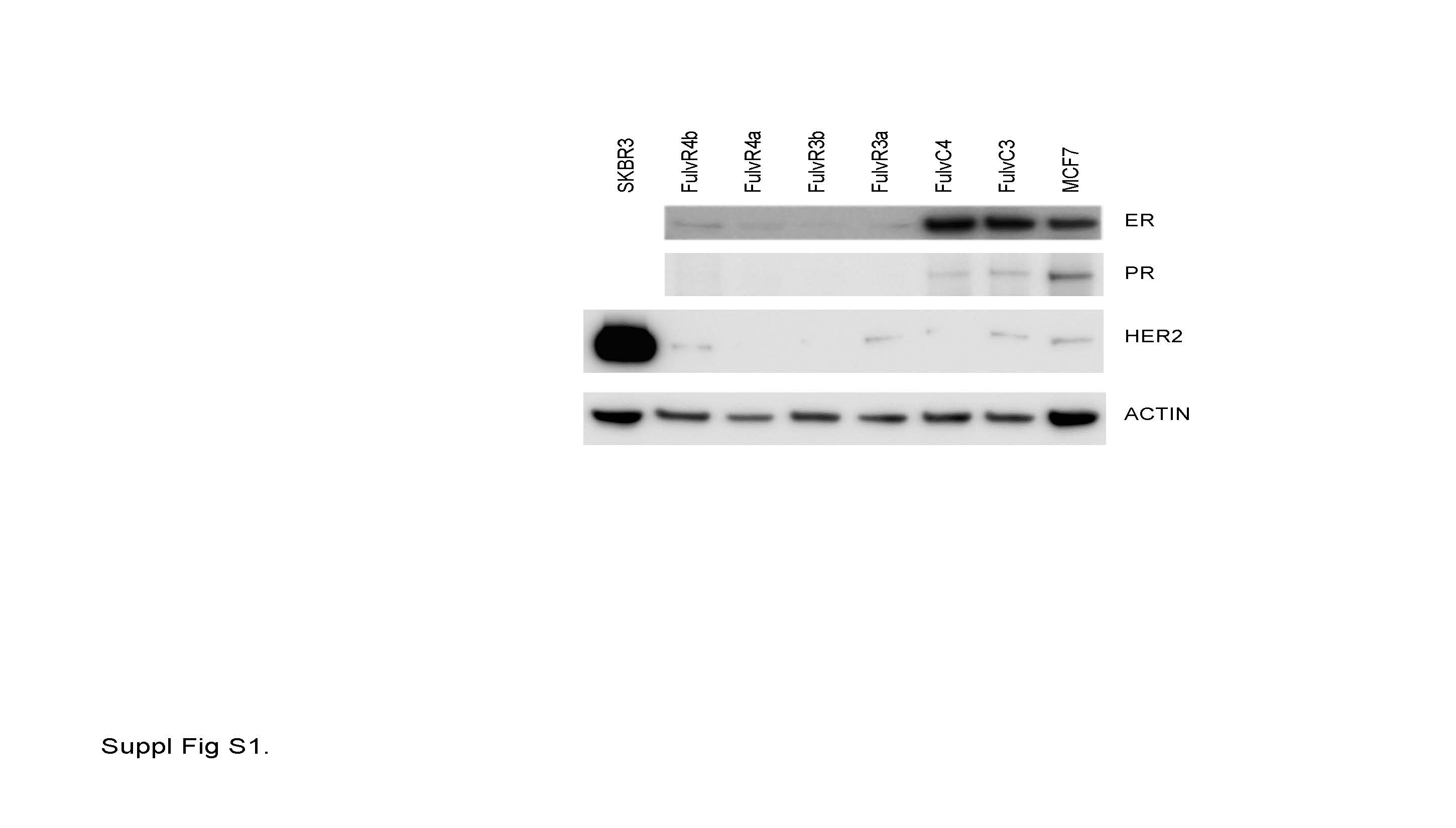

Supplement: Figure S1 — Relative expression of estrogen receptor (ER), progesterone receptor (PR), and HER2 in SKRB3, MCF-7 and a selection of its sub-lines. Actin is shown as loading control. [file Image_1.JPEG]

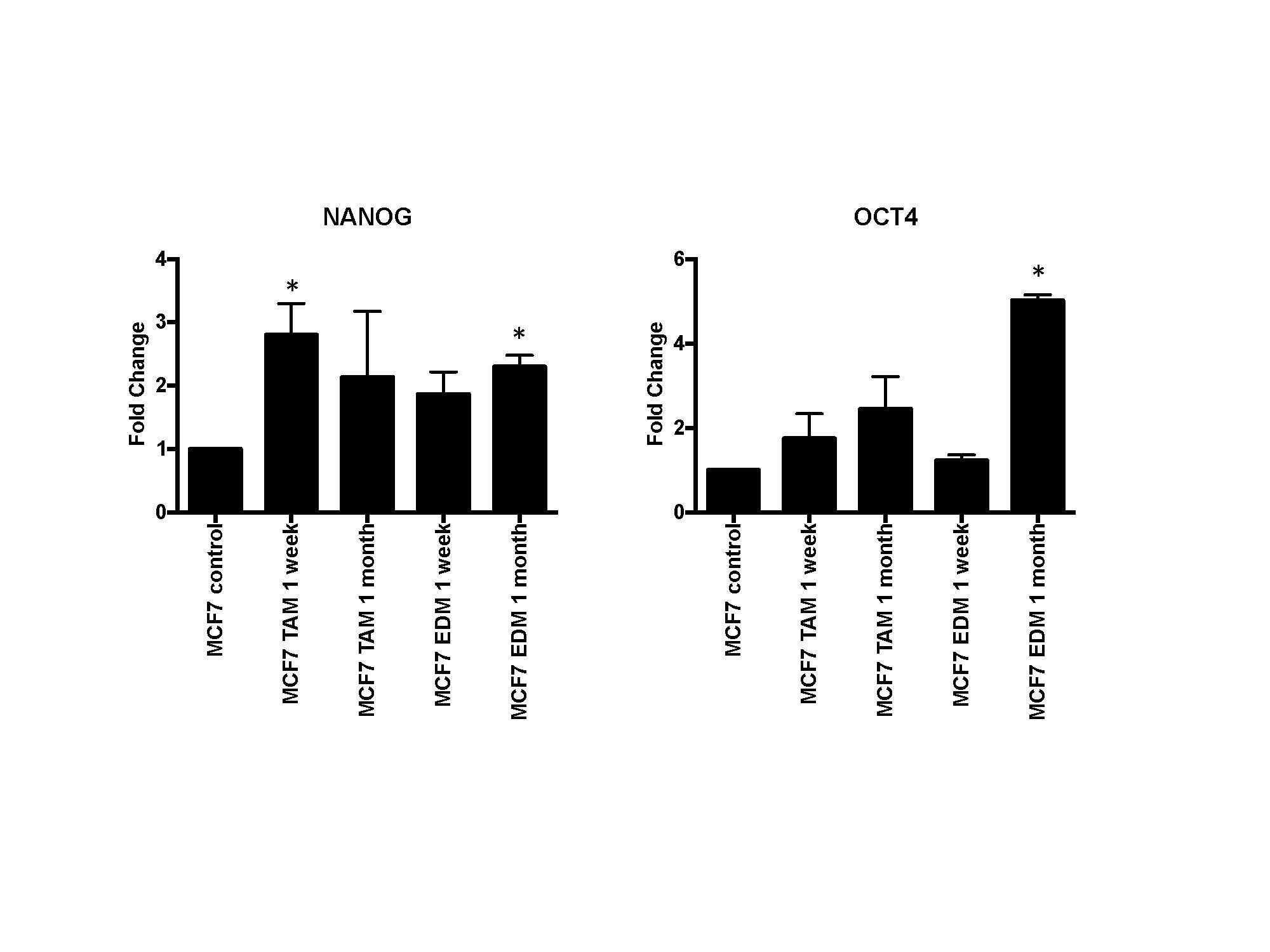

Supplement: Figure S2 — Increased expression of NANOG and OCT4 in MCF-7 breast cancer cells in 4-hydroxytamoxifen or estrogen-deprived treatment. The expression of (A) NANOG and (B) OCT4 relative to MCF-7 control measured by qRT-PCR in MCF-7 breast cancer cells either exposed to 4-hydroxytamoxifen (100 nM) or grown in estrogen-deprived medium (EDM) for 1 week and 1 month, relative to the MCF-7 parental line, *p < 0.05. [file Image_2.JPEG]

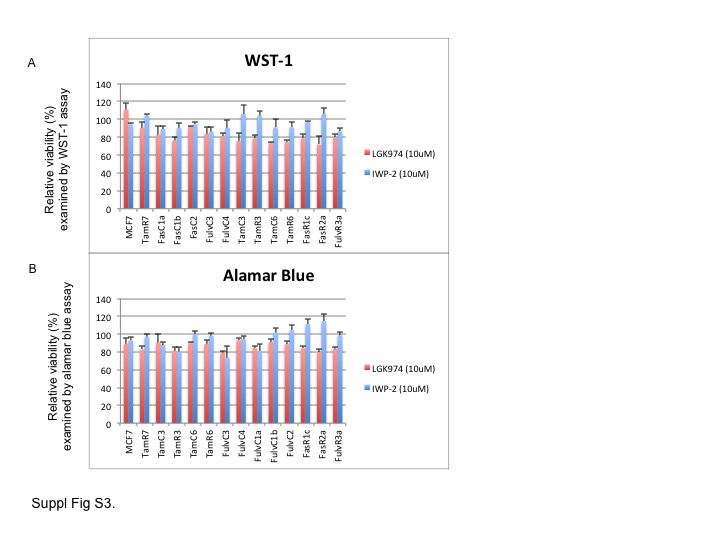

Supplement: Figure S3 — Relative viability of LGK974 and IWP-2 among breast cancer endocrine-resistant cell lines. Viability was measured by (A) WST-1 and (B) Alamar blue. Results are shown as the mean ± SE from duplicate experiments. [file Image_3.JPEG]
